# Diverse Chromatin Remodeling Genes Antagonize the Rb-Involved SynMuv Pathways in C. elegans

**DOI:** 10.1371/journal.pgen.0020074

**Published:** 2006-05-19

**Authors:** Mingxue Cui, E. Bridget Kim, Min Han

**Affiliations:** Howard Hughes Medical Institute and Department of Molecular, Cellular, and Developmental Biology, University of Colorado, Boulder, Colorado, United States of America; Huntsman Cancer Institute, United States of America

## Abstract

In *Caenorhabditis elegans,* vulval cell-fate specification involves the activities of multiple signal transduction and regulatory pathways that include a receptor tyrosine kinase/Ras/mitogen-activated protein kinase pathway and synthetic multivulva (SynMuv) pathways. Many genes in the SynMuv pathways encode transcription factors including the homologs of mammalian Rb, E2F, and components of the nucleosome-remodeling deacetylase complex. To further elucidate the functions of the SynMuv genes, we performed a genome-wide RNA interference (RNAi) screen to search for genes that antagonize the SynMuv gene activities. Among those that displayed a varying degree of suppression of the SynMuv phenotype, 32 genes are potentially involved in chromatin remodeling (called SynMuv suppressor genes herein). Genetic mutations of two representative genes *(zfp-1* and *mes-4)* were used to further characterize their positive roles in vulval induction and relationships with *Ras* function. Our analysis revealed antagonistic roles of the SynMuv suppressor genes and the SynMuv B genes in germline-soma distinction, RNAi, somatic transgene silencing, and tissue specific expression of *pgl-1* and the *lag-2/Delta* genes. The opposite roles of these SynMuv B and SynMuv suppressor genes on transcriptional regulation were confirmed in somatic transgene silencing. We also report the identifications of ten new genes in the RNAi pathway and six new genes in germline silencing. Among the ten new RNAi genes, three encode homologs of proteins involved in both protein degradation and chromatin remodeling. Our findings suggest that multiple chromatin remodeling complexes are involved in regulating the expression of specific genes that play critical roles in developmental decisions.

## Synopsis

In animal cells, DNA and genes are packed into a structure called chromatin. Chromatin-modifying protein complexes play a critical role in the regulation of gene expression. These complexes can alter the chemical and structural properties of the chromosome leading to either the repression or activation of gene expression. How these different complexes coordinate to regulate animal development remains to be explored. Several developmental processes in the nematode Caenorhabditis elegans present excellent model systems to study the functions of chromatin modifications. Using a genome-wide screen, the authors have identified 32 genes that encode potential chromatin-modifying proteins that antagonize the function of another set of transcription regulators including homologs of the mammalian Rb tumor suppressor and components of other chromatin-modifying complexes. The antagonistic roles of these two sets of genes have been observed in a variety of cellular and developmental processes, including organ development and expression of genes in particular tissues. This work indicates that multiple chromatin-modifying complexes are involved in maintaining proper expression of many genes that are critical for precise developmental decisions. Studies on these worm genes should shed light on the roles of the mammalian counterparts in development and related human diseases.

## Introduction

In the wild-type Caenorhabditis elegans hermaphrodite, the vulva is formed from the descendants of three of six vulval precursor cells (VPCs) [1,2]. Each VPC is initially equivalent in developmental potential. An epidermal growth factor–like signal from the gonad activates the receptor tyrosine kinase (RTK)/Ras/mitogen-activated protein kinase (MAPK) signaling pathway in three central VPCs, inducing them to adopt vulval cell fates [1,2]. A large group of genes, collectively called synthetic multivulva (SynMuv) genes, antagonize this RTK/Ras/MAPK signaling activity [3,4]. The SynMuv genes fall into three subgroups: classes A, B, and C. While a single loss-of-function *(lf)* mutation in each class does not result in obvious vulval induction defects, a combination of mutations in two classes gives rise to a robust Muv phenotype [3,5–7]. Cloning two of the SynMuv A genes, *lin-15A* and *lin-8,* has revealed that these encode nuclear proteins, suggesting that they may play a role in transcription regulation [8–10]. Many of the class B and C SynMuv genes encode transcription factors, including those homologous to components of the mammalian RB/E2F protein complex and chromatin-remodeling complexes (e.g., the nucleosome-remodeling deacetylase [NuRD] complex and the NuA4 [nucleosomal acetyltransferase of histone H4] complex) [4,7,11–18]. Previous studies on homologous proteins indicated that many SynMuv proteins likely function via transcriptional repression. For example, the NuRD complex represses transcription through nucleosomal remodeling and deacetylase activities. Recently, *Drosophila* RB/E2F-containing complexes (dREAM and Myb-MuvB complexes), which contain homologs of multiple SynMuv B proteins, have been shown to act as transcriptional repressors [19–21].

Besides their redundant roles in repressing vulval induction, the SynMuv B genes are also involved in multiple cellular functions during development. These roles include regulation of cell divisions and proliferation [22,23], regulation of context-dependent transcription silencing of transgenes in somatic cells [11], RNA interference (RNAi) [24], expression of *lag-2* in the intestine [18,25], and other developmental functions such as regulation of pharyngeal morphogenesis and larval development [26,27].

Chromatin remodeling plays a crucial role in regulating gene transcription. There are several ways that chromatin structure can be modulated [28]. First, the positioning of nucleosomes on DNA can be disrupted and reconfigured by ATP-dependent remodeling complexes. Second, posttranslational modifications by histone acetylases, deacetylases, and methyltransferases can generate localized distinct chromosomal domains by recruiting diverse chromatin-binding protein complexes. Third, the composition of nucleosomes can be modified by replacing major histones with variants. Most studies on chromatin modifications have been performed in yeast and mammalian cell cultures, and little is known about the functions of various chromatin-remodeling complexes during animal development. Studies of C. elegans vulval induction and other cellular and developmental processes provide us an opportunity to identify specific roles of these complexes in animal development.

To identify genes that antagonize the activity of SynMuv genes in regulating animal development, we have carried out a genome-wide RNAi screen. In this paper, we present the identification and characterization of a number of SynMuv suppressor genes that are involved in chromatin remodeling with respect to their roles in vulval induction, germline-soma distinction, germline and somatic transgene silencing, RNAi, and tissue-specific regulation of the expressions of the *pgl-1* and the *lag-2* genes.

## Results

### A Set of Chromatin Remodeling Genes Antagonize the SynMuv Gene Activities in Vulval Differentiation

A genome-wide feeding RNAi screen [29] was carried out to identify suppressors of the Muv phenotype of *lin-15AB(n765).* Among the 16,757 genes represented in the RNAi feeding library (out of the currently predicted 19,429 genes in C. elegans), we have identified more than 70 candidate genes. In three independent retests, RNAi of these candidate genes consistently suppressed the Muv phenotype of *lin-15AB(n765).* The sequence data of these identified genes were subsequently inspected for domain structures, as well as for homology to genes in *Homo sapiens, Drosophila melanogaster,* and Saccharomyces cerevisiae. The genes we identified include those that may function in RTK/Ras/MAPK and Notch signaling, cell fusion or migration, chromatin remodeling, dosage compensation, and other cellular processes. In this study, we focus on 32 candidate genes (called SynMuv suppressor genes herein) that are potentially involved in chromatin remodeling (Tables 1 and S1). Except for the six genes that have been recently identified as the SynMuv suppressors [24], 26 of the 32 genes were previously unknown for such a role. To investigate the allele specificity for suppression, we performed RNAi of these 32 genes in two other SynMuv mutants, *lin-36(n766); lin-15A(n767)* and *lin-8(n111); lin-15B(n374).* RNAi of all 32 genes significantly suppressed the Muv phenotype of at least one of these two strains (Table S2).

**Table 1:**
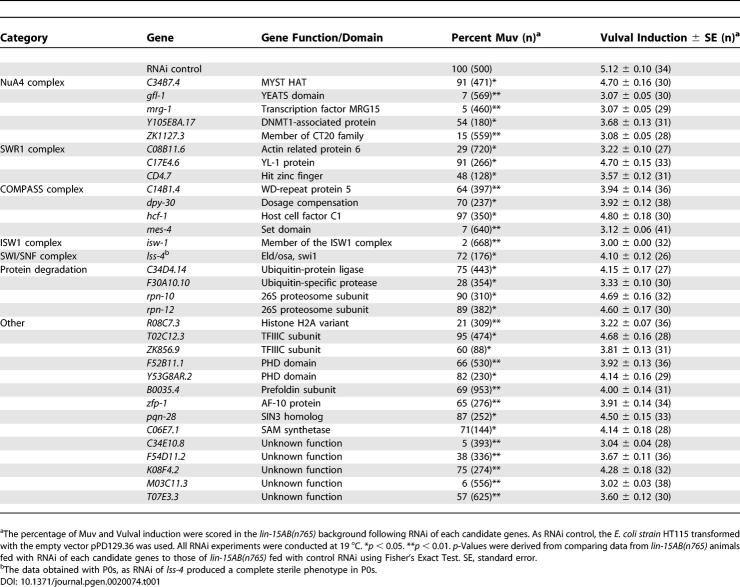
A Genome-Wide RNAi Screen Identified 32 SynMuv Suppressor Genes That Are Potentially Involved in Chromatin Remodeling

Among the 32 SynMuv suppressor genes, 14 encode proteins that are homologous to components of several known yeast and human chromatin remodeling complexes, such as NuA4 [30,31], SWR1 [32–34], COMPASS/MLL[35,36], ISW1[37], and SWI/SNF [38] complexes (Tables 1 and S1). Based on what are known to the chromatin remodeling complexes of their yeast homologs, these genes are further categorized into five groups. Proteins encoded by two genes in the NuA4 complex group, *mrg-1* and *ZK1127.3,* have been shown to directly bind each other in a yeast two-hybrid assay [39], implicating that they may function in the same complex in C. elegans. The yeast homologs of GFL-1 and Y105E8A.17 proteins are shared components of the NuA4 and SWR1 complexes in yeast [40]. The SWR1 complex in *S. cecrevisiae* is required for depositing the histone H2A variant H2A.Z into euchromatin to maintain gene expression [32–34]. An SWR1-like complex might also be present in C. elegans and may play a role similar to that of its yeast counterpart. This hypothesis was supported by the finding of R08C7.3, a histone-variant H2A.Z, as a strong SynMuv suppressor (Table 1).

We also identified four factors required for protein ubiquitylation and degradation, including two of the 19S regulatory particle (RP) subunits of the 26S proteosome (RPN-10 and RPN-12) [41], an E3 ubiquitin-protein ligase (C34D4.14), and an ubiquitin protease (F30A10.10). This implicates that protein ubiquitylation and degradation are involved in vulval differentiation. However, a recent finding has brought to light a distinct nonproteolytic role of 19S RP in chromatin remodeling [42]. The yeast ortholog of C34D4.14 directly binds to the two subunits of the yeast 19S RP [43], implicating that C34D4.14 may regulate chromatin remodeling through interacting with 19S RP of the 26S proteosome. In addition, the yeast H2B ubiquitin protease Ubp8, a C. elegans homolog of F30A10.10, is required for mediating the histone H2B ubiquitylation and deubiquitylation [44]. Taken together, it suggests that these four genes may play a role in chromatin remodeling to regulate vulval differentiation in C. elegans.

Proteins encoded by 9 of the 32 SynMuv suppressor genes are predicted to have roles in chromatin remodeling based on their molecular characteristics of their homologs in yeast and/or the functional domains they have. As mentioned above, *R08C7.3* encodes a histone H2 variant, H2A.Z. *T02C12.3* and *ZK856.9* encode the subunits of the general RNA polymerase III transcription factor TFIIIC. TFIIICs have been shown to have intrinsic histone acetyltransferase (HAT) activity to alleviate chromatin-mediated transcriptional repression of the RNA polymerase III–dependent gene in yeast [45,46]. *F52B11.1* and *Y53G8AR.2* encode proteins with a zinc finger-like plant homeodomain (PHD), which is found in many nuclear proteins with chromatin as their substrate [47]. *B0035.4* encodes a homolog of the yeast Gim3p. Gim3p, a component of the prefoldin complex, has been shown to function redundantly with components of the SWR1 complex in yeast [33]. Mammalian ortholog of ZFP-1 binds to the GFL-1 ortholog GAS41, a component of human NuA4 complex [48]. *pqn-28* encodes a C. elegans homolog of yeast Sin3p, which is well known for its function in chromatin remodeling [24,49]. In agreement with the possible involvement of histone methyltransferase in vulval development, RNAi of a S-adenosylmethionine synthetase (SAM) gene *(C06E7.1)* suppressed the SynMuv phenotype. There are five SAM synthetase genes, *C06E7.1, C06E7.3, C49F5.1, T13A10.11,* and *Y105C5B.12,* which encode proteins shared 99% homology. We expect that RNAi of *C06E7.1* would have cross-reactions with the other four genes.

The remaining five genes, *C34E10.8, F54D11.2, K08F4.2, M03C11.3,* and *T07E3.3,* encode proteins with unknown functions. Their genetic properties (see below for detail) are more similar to those of the known chromatin factors described above than to those of other SynMuv suppressors such as genes involved in RTK/Ras/MAPK signaling, cell fusion, or cell migration (unpublished data). This suggests that these five genes may also function in chromatin remodeling. We thus characterize them together with the known chromatin factors even though it is still possible that they do not act as transcriptional regulators.

### *zfp-1* and *mes-4* Promote Vulval Induction in SynMuv Mutants

**Figure 1:**
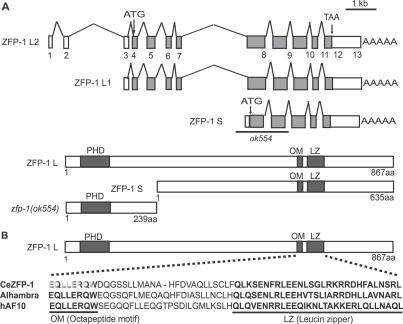
The *C. elegans zfp-1* Gene (A) Schematic illustration of the transcripts and proteins of *zfp-1* (http://www.wormbase.org). In *zfp-1(ok554)* mutant, the fifth and sixth exons were deleted and 2 nucleotides (CG) were inserted. Therefore, the short isoform, ZFP-1S, is unlikely to produce any proteins and the long isoform is expected to generate a truncated protein that lacks the OM-LZ motifs in the mutant. (B) Diagram of the functional motifs of the long isoform of ZFP-1 and the sequence alignment of the OM and LZ motifs. C. elegans ZFP-1 and its homologs from human (hAF10) and *Drosophila* (Alhambra) are compared.

To validate the results of the RNAi screen and better characterize the functions of the SynMuv suppressor genes, we carried out further analysis of *zfp-*1 and *mes-4* using genetic mutations. *zfp-1* is a C. elegans ortholog of the human *AF10* gene [50]. AF10 directly binds to GAS41 (a component of human NuA4 complex) and hDOT1 (a H3 K79 methyltransferase) [48,51]. ZFP-1 shares the highly conserved motifs of the AF10 protein family including the amino-terminal PHD finger, an octapeptide motif (OM), and a leucine zipper motif (LZ) in its carboxy terminus (Figure 1). The OM-LZ motifs have been shown to be required for leukemic transformation by the MLL-AF10 fusion protein [52]. The *zfp-1* gene encodes three different transcripts, *zfp-1 L1, zfp-1 L2,* and *zfp-1 S,* based on expressed sequence tag data (http://www.wormbase.org; Figure 1). Both *zfp-1 L1 and zfp-1 L2* are predicted to encode the larger protein (867 amino acids) bearing the PHD finger and the OM-LZ motifs. *zfp-1 S* encodes a shorter protein (635 amino acids) that lacks the PHD finger but retains the OM-LZ motifs. We obtained a deletion mutant affecting the *zfp-1* gene from the International C. elegans Gene Knockout Consortium. This mutation, *ok554,* removes 1,677 base pairs from the *zfp-1* locus and is predicted to generate a truncated protein that consists of the first 240 amino acids of the larger protein, resulting in the lack of the essential OM-LZ motifs (Figure 1). *zfp-1(ok554)* animals were viable, although they exhibited some developmental defects such as slow growth, protruding vulva, and a reduced brood size (unpublished data), which were also associated with *zfp-1(RNAi).* Consistent with the RNAi experiment, *zfp-1(ok554)* significantly reduced the Muv phenotype of *lin-15AB(n765)* from 100% to 62% at 19 °C, and from 98% to 19% at 18 °C (Table 2). Furthermore, *zfp-1(ok554)* strongly suppressed the Muv phenotype of *lin-35(n745); lin-8(n111)* at 19 °C (Table 2).

**Table 2:**
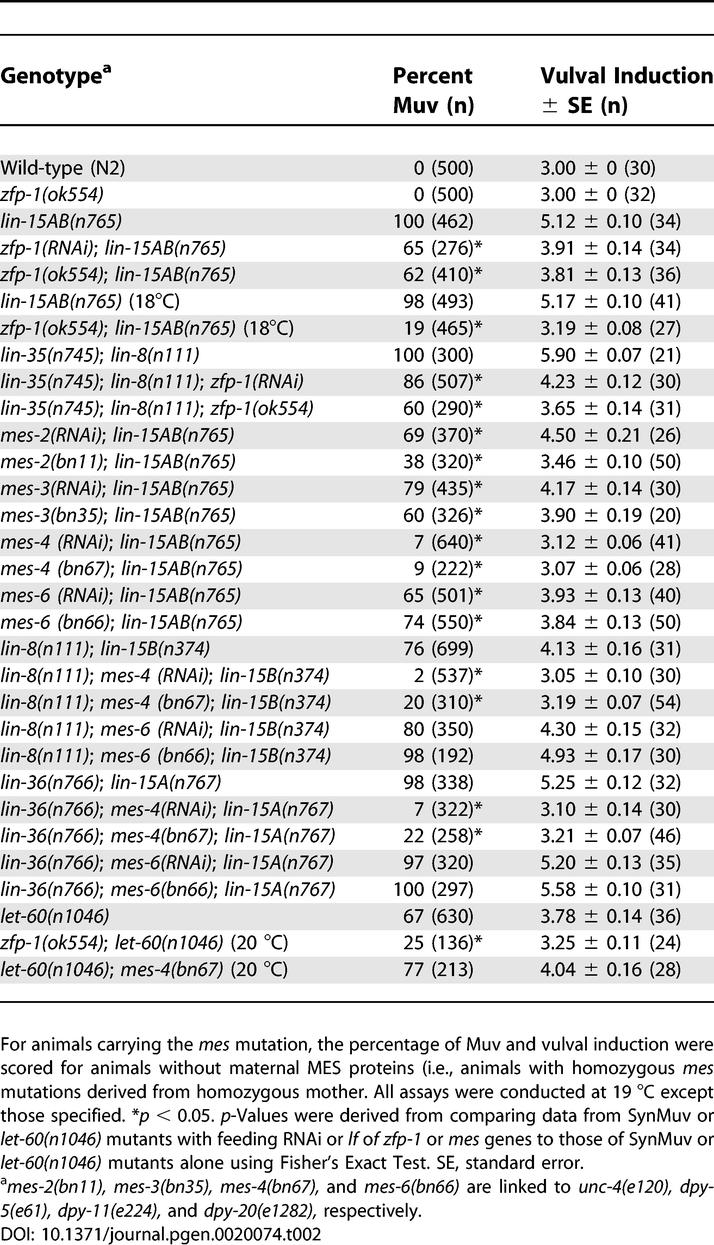
Genetic Interactions of *zfp-1* and *mes* Genes with SynMuv and *ras* Genes

*mes-2, mes-3, mes-4,* and *mes-6* genes were identified as maternal-effect sterile genes essential for germline viability [53]. It has been proposed that MES-2/3/6 complex, together with MES-4, are involved in X-chromosome silencing, and that MES-4 functions to exclude repressors from the autosomes [54]. Initially, we identified the RNAi of all four *mes* genes that suppressed the Muv phenotype of *lin-15AB(n765)* from the RNAi screen. However, only *mes-4* could suppress the other tested SynMuv mutants by RNAi. We then carried out a genetic test using the *lf* mutations of *mes-4* and *mes-6*. We confirmed that *mes-4(lf),* but not *mes-6(lf),* suppressed the Muv phenotype of all tested SynMuv mutants in the absence of the maternal MES proteins contribution (Table 2, unpublished data). MES-4 is homologous to the human MLL and the *Drosophila* trithorax proteins, which are histone methyltransferases maintaining active gene expression during development. In regard to a previous finding that shows MES-4 associated specifically with autosomes [54], MES-4 may positively regulate the expression of genes on autosomes by modifying chromatin, and thus antagonizing the repression function of the SynMuv genes during vulval development.

The allele-specific suppression of the Muv phenotype of *lin-15AB(n765)* by RNAi of *mes-2, mes-3,* and *mes-6* genes can be explained by their roles in X-chromosome dosage compensation [55]. The Muv phenotype of *lin-15AB(n765)* has been previously shown to be suppressed by dosage compensation defects [56,57], which is consistent with the X-chromosomal location of the *lin-15A* and *lin-15B* genes.

Interestingly, we found *mes-6(bn66)* enhanced the Muv phenotype of *lin-8(n111); lin-15B(n374)* from 76% to 98% Muv. This result suggests that *mes-6* may play a role in the SynMuv gene–related transcription repression. Such a repression function is consistent with the facts that the MES-2/MES-3/MES-6 complex keeps the X-chromosome repressed during germline development [54], and that *Drosophila* enhancer of zeste and extra sex combs, homologs of MES-2 and MES-6, respectively, are members of the Polycomb group of transcriptional repressors [58].

### The Relationship between SynMuv Suppressors and Ras

The SynMuv genes are known to act negatively on RTK/Ras/MAPK–mediated vulval induction [2]. Gain-of-function *(gf)* mutations in *let-60/Ras* cause a Muv phenotype. To investigate the relationship between identified SynMuv suppressors and the Ras pathway, we performed RNAi of each of the 32 candidate genes in *let-60(n1046 gf)* animals. We found that the Muv phenotype of *let-60(n1046)* could not be suppressed by RNAi of any of these genes (unpublished data). Consistent with the RNAi results, we observed no suppression of the Muv phenotype of *let-60(n1046)* by the *mes-4(lf)* mutation mentioned above (Table 2). These results suggest that most of the SynMuv suppressor genes may function upstream of the Ras pathway. However, it is possible that the RNAi of some of these genes were too weak to suppress the Muv phenotype of *let-60(n1046)* even though it is effective in suppressing the Muv phenotype of *lin-15AB(n765).* In contrast to *mes-4(lf), zfp-1(ok554)* displayed a significant suppression of the Muv phenotype of *let-60(n1046)* (Table 2), suggesting that *zfp-1* may have an activity downstream or in parallel to *let-60/Ras.* This does not exclude that *zfp-1* also acts with *mes-4* on a target upstream of *let-60/Ras.* The different effects of the *mes-4* and *zfp-1* mutations on *let-60(gf)* suggest that they act at two different steps to regulate vulval induction.

### SynMuv Suppressor Genes Are Required for the Somatic Expression of the Germline-Specific Gene *pgl-1*

**Figure 2:**
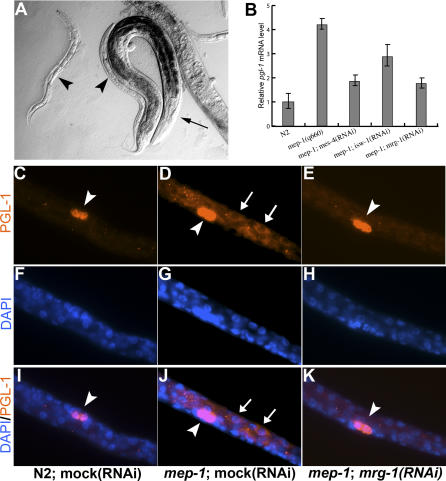
The SynMuv Suppressor Genes Antagonize the SynMuv B Genes on Germline-Soma Distinction and *pgl-1* Expression (A) The early larval arrest phenotype associated with the SynMuv B mutation *mep-1(lf)* was rescued by RNAi of *mrg-1.* The growth-arrested L1 larvae of *mep-1(lf)* (arrowheads) and the rescued adults of *mep-1(lf)* treated with *mrg-1(RNAi)* (arrow) are indicated. RNAi of 11 other SynMuv suppressor genes displayed a varying degree of rescue (Table 3). (B) Quantification of relative mRNA levels of the germline specific gene *pgl-1* in animals with the genotype indicated. Only three strong larval arrest rescuers, *mes-4, mrg-1,* and *isw-1,* were assayed here. *rpl-26* was used as the internal reference. Mean values and ranges of the *pgl-1*/*rpl-26* ratios based on three qRT-PCR trials are shown. (C–K) Immunostaining using an anti–PGL-1 antibody shows that *mep-1(q660)* and *mrg-1(RNAi)* have opposite effects on ectopic expression of *pgl-1* in soma. (C–E) Immunofluoresence micrographs of L1 larvae stained with an anti-PGL antibody (red). The genotype of each animal is indicated at the bottom of the corresponding column. (F–H) DAPI staining (blue) of the same animals to indicate nuclei. (I–K) The merged micrographs. White arrowheads indicate germline cells. Arrows indicate the hypodermal cells. The *mep-1* mutation (D, G, and J) as well as several other SynMuv B genes (not shown, see text) caused ectopic staining of PGL-1 in somatic cells such as hypodermal cells. This ectopic staining was suppressed by *mrg-1*(RNAi) (E, H, and K) and RNAi of two other SynMuv suppressor genes (*isw-1* and *mes-4*)(not shown, text). Bar: 100 μm for A, 10 μm for C-K.

*mep-1* encodes a zinc-finger protein that interacts with LET-418/Mi-2 of the NuRD complex to function in the SynMuv B pathway as well as in maintaining germline-soma distinctions in C. elegans [16]. *lf* mutations in *mep-1* cause a first or second larval (L1/L2) lethality phenotype and ectopic expression of germline-specific genes, such as *pgl-1,* in somatic cells. It has also been shown that mutations in *mes-2, mes-3, mes-4,* and *mes-6* result in a partial suppression of ectopic *pgl-1* expression and a partial rescue of the larval lethality of *mep-1(lf)* [16]. Because *mes-4* is one of the strong SynMuv suppressor genes, we investigated whether RNAi of the other SynMuv suppressor genes could rescue the *mep-1* larval lethality. We found that RNAi of 12 of the 32 SynMuv suppressor genes, including *mes-4,* rescued the L1/L2 larval lethality of *mep-1(q660)* at 25 °C (Table 3, Figure 2A, and unpublished data). RNAi of *isw-1, mes-4,* and *mrg-1* displayed the strongest rescue, resulting in 100% viable adults (Table 3, Figure 2A, and unpublished data).

**Table 3:**
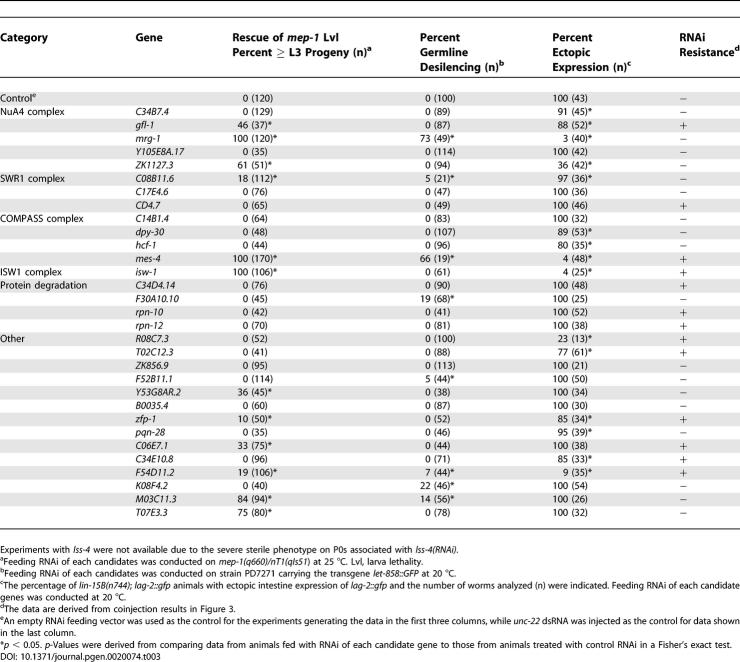
SynMuv Suppressor Genes Play a Role in Rescuing *mep(lf)* Larval Lethality, Germline Transgene Silencing, RNAi and the Ectopic Expression of *lag-2* in Intestine

**Figure 3:**
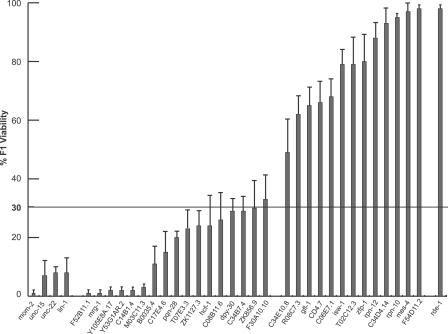
Identification of Genes Required for Robust RNAi WT (N2) young adult hermaphrodites were co-injected with 300 ng/μl of each candidate dsRNA and 75 ng/μl of *mom-2* dsRNA. *rde-1(RNAi)* was used as a positive control, while *unc-15(RNAi), unc-22(RNAi),* and *lin-1(RNAi)* were used as negative controls. The horizontal line indicates the cut-off for viability (>30%) that was used to define the positive genes reported in Table 3. All positive genes displayed significant differences from the negative controls with *p* < 0.01 (*t* test). The positive candidates include three genes *(gfl-1, mes-4,* and *zfp-1)* that were previously identified by Dudley et al. using the same approach [50]. Error bars represent standard error.

We then further investigated the effects of *mep-1(q660)* and RNAi of three SynMuv suppressor genes on the expression of *pgl-1* in L1 larvae using real-time RT-PCR. The mRNA level of *pgl-1* in *mep-1(q660)* animals was increased by about 4-fold compared to that in wild-type (WT) animals (Figure 2B). The mRNA level in *mep-1(q660)* mutant was down-regulated in animals treated with RNAi of *isw-1, mes-4,* and *mrg-1* (Figure 2B). To confirm that the down-regulation of *pgl-1* resulted from the suppression of the ectopic expression in somatic cells, we immunostained *mep-1(q660)* animals treated with or without RNAi of *isw-1, mes-4,* and *mrg-1* using an anti-PGL-1 antibody (gift from S. Strome). The suppression of ectopic expression of PGL-1 in the somatic cells of *mep-1(q660)* animals treated with RNAi of *isw-1, mes-4,* and *mrg-1* was evident (Figure 2C–2K). These data indicate that the expression changes obtained from real-time RT-PCR are due to the ectopic expression changes in the soma. It is important to note that the disappearance of PGL-1 staining in somatic cells of *mep-1(lf)* animals upon inactivation of *isw-1, mes-4,* and *mrg-1* was in accordance with the disappearance of germline cell–like somatic cells (unpublished data). This observation indicates that the soma to germline transformation associated with the *mep-1(lf)* mutant depends on activities of these SynMuv suppressors.

In addition, we immunostained three other SynMuv B mutants, *lin-9(n112), lin-15B(n744),* and *lin-35(n7450),* each treated with or without RNAi of *isw-1, mes-4,* and *mrg-1.* We observed ectopic expression of PGL-1 in somatic cells of all three SynMuv B mutants and the suppression of ectopic expression when animals were treated with RNAi of *isw-1, mes-4,* and *mrg-1* (unpublished data). These results are consistent with a recent report that the ectopic expression of *pgl-1* in somatic cells in a number of SynMuv B mutants and can be suppressed by *mes-4(RNAi)* [24]. The data here suggest that the multiple SynMuv suppressor genes are required for ectopic expression of *pgl-1* in the soma, which is normally repressed by a number of SynMuv B genes, including *mep-1, lin-35/Rb, lin-9, lin-15B,* etc.

### SynMuv Suppressor and SynMuv B Genes Play Opposite Roles in RNA Interference

During the genome-wide RNAi screen, we noticed that *lin-15AB(n765)* enhanced the effect of RNAi for many genes. To find out whether other SynMuv mutants are also more sensitive to RNAi than WT animals, we assayed a number of SynMuv A and B mutants by feeding RNAi of five genes, *lin-1, hmr-1, dpy-13, rol-3,* and *unc-33,* which had been previously used to test the efficiency of RNAi for the *rrf-3* mutant [59]. Except for *lin-36(n766),* SynMuv B mutants *lin-9(n112), lin-15B(n744), lin-35(n745), lin-37(n758),* and *tam-1(cc567*) displayed an increase in RNAi sensitivity (Table S2). In particular, the *lin-9(n112)* mutant displayed the strongest RNAi-sensitizing effect similar to that of the *rrf-3(pk1426)* and *eri-1(mg366)* mutants, which are known as the RNAi-sensitive mutants [59,60]. In contrast, we found that the SynMuv A mutants *lin-8(n111)* and *lin-38(n751)* displayed no change in RNAi sensitivity (Table S2). These findings agree with a recent report that a number of SynMuv B genes, including *lin-35/Rb,* play a negative role in RNAi [24].

Among the 32 SynMuv suppressor genes we identified, six of these *(mes-4, gfl-1, zfp-1, pqn-28, M03C11.3,* and *ZK1127.3)* have previously been shown to play a positive role in RNAi [50,61]. Recently, these genes have been independently identified as SynMuv suppressors using a different approach [24]. To find out whether other SynMuv suppressor genes are also involved in the RNAi pathway, we co-injected WT animals with double-stranded RNA (dsRNA) of each of the 32 SynMuv suppressor genes together with dsRNA of *mom-2,* a gene essential for viability [50,61]. The survival of progeny indicates that RNAi of the candidate gene enables animals to overcome the lethality of *mom-2(RNAi).* Worms co-injected with dsRNA of a positive control gene, *rde-1,* and dsRNA of *mom-2* showed 98% of survival, indicating strong protection from *mom-2* dsRNA induced lethality by *rde-1(RNAi).* In contrast, co-injection with dsRNAs of *unc-*15*, unc-22,* or *lin-1* that are dispensable for RNAi or injection with *mom-2* dsRNA alone resulted in a low percentage of survival (Figure 3). Co-injection of dsRNAs of 13 of the 32 SynMuv suppressor genes significantly rescued the lethality caused by injection of *mom-2* dsRNA (Table 3 and Figure 3), suggesting that these genes are required for robust RNAi. Except for *mes-4, gfl-1,* and *zfp-1,* which have been reported earlier as RNAi factors [50,61], the roles of ten of the 13 genes in RNAi were not previously known. These results suggest that SynMuv genes and SynMuv suppressors play opposite roles in the RNAi process.

Notably, three genes related to protein degradation *(C34D4.14, rpn-10,* and *rpn-12)* were determined to be involved in RNAi in the above assay (Table 3 and Figure 3). It suggests that RNAi mechanism involves protein degradation and/or chromatin remodeling mediated by 19S RPs and E3 ubiquitin-protein ligase.

*pgl-1* has been shown to be required for germline RNAi [62] and is ectopically expressed in the soma of SynMuv B mutants ([16,24] and this work). We asked whether a *pgl-1* null mutation, *ct131* [63], could suppress the RNAi-sensitizing effect of SynMuv B mutants. Three double mutants, *lin-35(n745)*; *pgl-1(ct131), pgl-1(ct131)*; *lin-15B(n744),* and *lin-9(n112)*; *pgl-1(ct131),* which were treated with RNAi of *rol-3,* still produced obvious roller progeny similar to the SynMuv B single mutants, indicating that *pgl-1* had no effect on the RNAi-sensitizing effect associated with these three SynMuv B mutants. This result suggests that *pgl-1* is not necessary for RNAi sensitivity of the SynMuv B mutants.

We further investigated if *lin-35(n745)* up-regulated the expression of any of 11 RNAi pathway genes *(dcr-1, ego-1, mut-7, mut-14, mut-16, rde-1, rde-2, rde-3, rde-4, rha-1,* and *rrf-1).* The mRNA levels of all 11 genes were compared between WT animals and the *lin-35/Rb* mutant by real-time RT-PCR. No significant changes were observed for any of the 11 transcripts in the *lin-35/Rb* mutant (Figure S1), suggesting that effects of SynMuv B mutations on RNAi sensitivity is rather specific on a certain, unknown component(s) of the RNAi machinery.

### The Antagonistic Role of the SynMuv Suppressors and the SynMuv B Genes in Somatic Transgene Silencing Was Achieved through Transcription Regulation

**Figure 4:**
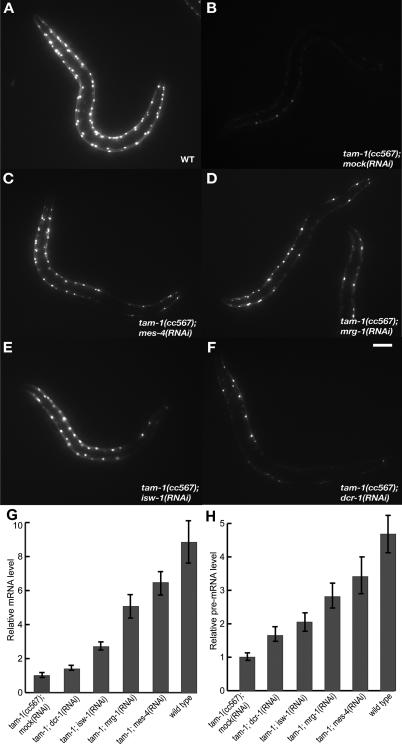
Somatic Trangene Silencing by *tam-1(lf)* Is Desilenced by RNAi of the SynMuv Suppressor Genes and Dicer (A–B) *myo-3* promoter driving-GFP reporters *(myo-3::Ngfp-lacZ*, *myo-3::Mtgfp)* displayed significant transgene silencing in the somatic tissues (mainly the muscles) in the *tam-1(cc567)* mutant (B), but not in the WT animals (A) [11]. (C–F) RNAi of the SynMuv suppressors, *mes-4* (C), *mrg-1* (D), and *isw-1* (E) as well as a non-SynMuv suppressor gene, *dcr-1* (F), restored GFP expression in the *tam-1(cc567)* mutant. *dcr-1* encodes the C. elegans Dicer protein that is required for *RNAi* [81,82]. Bar: 100 μm. (G–H) The relative levels of *gfp/lacZ* of a transgene *ccIs4251 (myo-3::Ngfp-lacZ*, *myo-3::Mtgfp)* [11] were measured by qRT-PCR in various genetic background indicated. The level of *ama-1* mRNA, encoding an RNA PolII, was used as the internal reference. Mean values and ranges of the *lacZ/ama-1* ratios based on three qRT-PCR trials are shown.

A number of SynMuv B genes, including *tam-1, lin-35, lin-9, lin-15B, lin-51,* and *lin-52,* are known to be involved in the modulation of context-dependent transgene silencing in somatic cells [11]. *lf* of any of these SynMuv B genes results in the hypersilencing of numerous highly repeated transgenes in somatic cells. To assess whether this transgene silencing is a transcriptional or a posttranscriptional event, we performed real-time RT-PCR to investigate the levels of pre-mRNA and mRNA of a transgene *ccIs4251 (myo-3::Ngfp-lacZ, myo-3::Mtgfp)* in *tam-1(cc567)* using the method developed by Grishok et al. [64]. *tam-1,* a SynMuv B gene, encodes a RING finger/B-Box factor that has been shown to be involved in context-dependent gene silencing [11]. We observed that the levels of *gfp/lacZ* pre-mRNA and mature mRNA in *tam-1* animals decreased by 3-fold and 10-fold, respectively, compared to those in WT animals (Figure 4G–4H). These data indicate that transgene silencing in the *tam-1* mutant likely occurs at the pre-mRNA stage and may share the same mechanism as the previously described RNAi-induced transcriptional gene silencing [64].

We next examined whether RNAi of the SynMuv suppressors could revert the somatic transgene silencing in the *tam-1(cc567)* mutant. Among the 32 SynMuv suppressor genes, we found that RNAi of *mes-4*, *isw-1, mrg-1, zfp-1,* and *C06E7.1* prevented transgene silencing in somatic tissues in the *tam-1(cc567)* mutant (Figure 4A–4E and unpublished data). RNAi of *dcr-1,* which encodes the only dicer protein in *C. elegans,* was also found to be capable of suppressing the somatic transgene silencing in the *tam-1* mutant (Figure 4F). To quantify the observed effects, we performed real-time RT-PCR to measure the pre-mRNA and mature mRNA levels of the transgene following injection RNAi of *dcr-1, mes-4, isw-1,* and *mrg-1* in the *tam-1 (cc567)* mutant. The levels of both pre-mRNA and mRNA of the transgene, *myo-3::gfp/lacZ,* were significantly increased following RNAi of these four genes (Figure 4G–4H). These results suggest that these SynMuv suppressor genes are required for somatic transgene silencing in the *tam-1* mutant and regulating transgene expression at the transcription level as the SynMuv B genes do.

### SynMuv Suppressor Genes Are Required for Germline Transgene Silencing and May Function in Germline Maintenance

In C. elegans, multiple-copy transgenes are commonly silenced in the germline. It depends on the four *mes* genes *(mes-2, mes-3, mes-4,* and *mes-6* [65]), *his-24* [66], the SynMuv B gene *hpl-2* [67], and a number of genes that function in the RNAi pathway and germline cosuppression [61,62].

**Figure 5:**
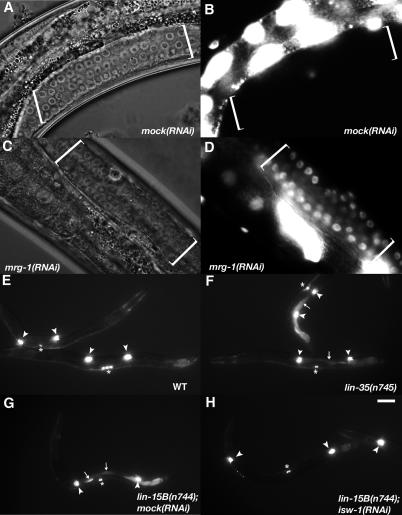
The SynMuv Suppressor Genes Are Required for Germline Transgene Silencing in WT Animals and for the *lag-2::gfp* Ectopic Expression in *lin-15B(n744)* (A–D) DIC (A) and (C) and GFP fluorescence (B) and (D) images of hermaphrodite germline from transgenic strain PD7271 that contains the multicopy *let-858::gfp* reporters [75]. Brackets indicate the region of germ cell nuclei. The transgene was silenced in WT, but desilenced in animals treated with *mrg-1(RNAi).* RNAi of seven other SynMuv suppressors also displayed a similar effect (Table 3). Bar: 10 μm. (E–H) GFP fluorescence images of mid-L4 larvae carrying the *lag-2::gfp* transgene. (E) WT animals display a strong expression of the transgene in distal tip cells (arrowheads) and the vulva (asterisks). (F–G) a strong ectopic expression of the transgene in the intestine (arrows) is seen in two SynMuv mutants. (H) RNAi of *isw-1* suppressed the ectopic expression of *lag-2::gfp* in the intestine of the *lin-15B(n744)* mutant, but not its expression in distal tip cells and vulval cells. RNAi of 14 other SynMuv suppressors also displayed a similar effect (Table 3). Bar: 100 μm.

We investigated whether the SynMuv suppressor genes are required for germline transgene silencing using the marker transgene *let-858::GFP* (PD7271), which is silenced in the germline but expressed in somatic tissues [65]. RNAi of eight of the 32 SynMuv suppressor genes resulted in desilencing of the transgenes in the germline (Table 3 and Figure 5A–5D), suggesting that they are required for inhibiting the expression of transgenes in the germline. These included six genes that have not been previously reported for such function: *mrg-1, C08B11.6, F30A10.10, F52B11.1, F54D11.2,* and *K08F4.2.* The molecular functions of the latter two genes are not known. However, as their genetic behaviors are more close to the potential chromatin remodeling genes, we propose that *F54D11.2* and *K08F4.2* may play a role related to chromatin remodeling.

It has been reported previously that mutations in the known germline silencing genes (the four *mes* genes, *his-24,* and *hpl-2*) cause sterile phenotypes and disrupt germline maintenance [65–67]. We observed that RNAi of the two new germline silencing genes, *mrg-1* and *F30A10.10,* displayed a completely sterile progeny phenotype, suggesting that they might be required for germline maintenance. The sterility of *mrg-1(RNAi)* has been reported and shown to be the consequence of a defect in mitotic proliferation of primordial germ cells [68,69].

### SynMuv Suppressor Genes Are Enriched in Germline Gene Clusters

The germline functions of the SynMuv suppressor genes are consistent with the distribution of most of these genes in germline gene clusters. Using TopoExpression Mountains devised by Kim et al. [70], we found that seven “mountains” were overrepresented (*p* < 0.05) by 32 SynMuv suppressor genes, whereas six “mountains” were overrepresented by the 33 SynMuv genes (Table 4). More strikingly, two germline-related mountains (mount 11 and mount 18), as well as the so-called “germline-enriched group,” were overrepresented by both the SynMuv genes and the SynMuv suppressor genes. Specifically, 16 SynMuv suppressor genes, *dpy-30, gfl-1, isw-1, mes-4, mrg-1, pqn-28, rpn-12, C08B11.6, C14B1.4, C17E4.6, C34E10.8, C46A5.9, CD4.7, F30A10.10, K08F4.2, M03C11.3, R08C7.3, B0035.4,* and *ZK856.9,* are in either mount 11 or the germline-enriched group [70]. RNAi of half of these genes, *isw-1, mes-4, mrg-1, pqn-28, rpn-12, F30A10.10, M03C11.3,* and *R08C7.3,* displayed a sterile phenotype.

**Table 4:**
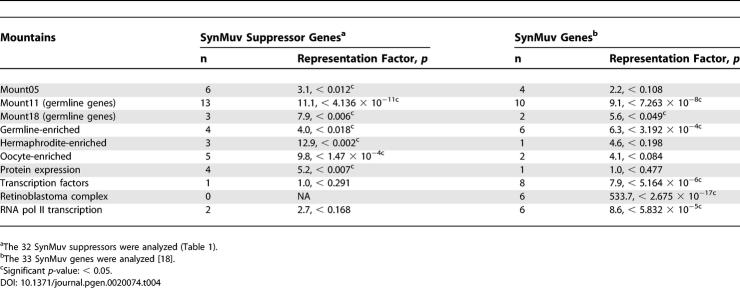
Representations of TOPO Expression Mountains by the SynMuv Suppressor Genes and the SynMuv Genes

### SynMuv Suppressor Genes Are Required for the Ectopic Expression of the *lag-2* Gene in SynMuv B Mutants

*lag-2* encodes a Delta*-*like ligand for the Notch receptors GLP-1 and LIN-12 that mediate multiple cell–cell interaction events in C. elegans [71]. In WT animals, *lag-2* is expressed in distal tip cells of the gonad to signal GLP-1 and controls germline proliferation. It has been known that a number of SynMuv genes transcriptionally repress the *lag-2::gfp* expression in the intestinal and epidermal cells [18,25]. Consistent with these previous reports in which RNAi of the SynMuv genes was being used, we found that a genetic mutation in *lin-15B* produced a strong ectopic *lag-2::gfp* expression in the intestinal cells (Figure 5E–5H and Table 3). To investigate whether the SynMuv suppressor genes are required for the ectopic expression of *lag-2* in the intestine of SynMuv B mutant, we performed RNAi of each of the 32 SynMuv suppressor genes on the *lin-15B* mutant carrying the *lag-2::gfp* transgene and scored for ectopic *lag-2::gfp* expression (Figure 5E–5H and Table 3). We observed that RNAi of 15 of the 32 SynMuv suppressors suppressed the ectopic reporter gene expression in the intestine. These data indicate that these SynMuv suppressors are required for the ectopic expression of *lag-2* in the intestine and may function as transcription activators. These results are consistent with the hypothesis that both the SynMuv suppressor genes and the SynMuv B genes act antagonistically in transcription regulation of common target genes.

## Discussion

### Chromatin Remodeling Genes Play Dual Roles in Vulval Development, Germline-Soma Distinction, and RNAi

A number of SynMuv B proteins are homologous to yeast, *Drosophila,* and/or human proteins that have been implicated in histone modification, nucleosome remodeling, and transcription repression. Some SynMuv B proteins are homologs of components of the NuRD complex that is well known to play a major role in transcription repression [4,13–15,17]. Recently, the *Drosophila* Myb-MuvB (also known as dREAM) complex, which contains homologs of the C. elegans SynMuvB gene products (lin-35/Rb, hda-1, lin-52, lin-61, lin-9, efl-1, and DPL-1) has been isolated as a transcription repressor complex from embryonic extracts [19,20]. Thus, it has been proposed that chromatin remodeling plays a repressive role on inductive signaling in vuval development in C. elegans [7,15]. Our findings revealed that chromatin remodeling plays a dual role in vulval development, both repressive and active. Taking the biochemical characterizations of their mammalian and yeast counterparts into consideration, the SynMuv suppressor genes we identified have links to transcription activation. Histone acetylation carried out by the NuA4 complex and/or the subunits of TFIIIC, the incorporation of H2A.Z into the euchromatin carried out by the SWR1 complex, and the histone H3 K4 tri-methylation carried out by the COMPASS and ISW1 complexes, are generally associated with transcription activation. Therefore, we propose that the SynMuv suppressor genes compete with the SynMuv genes at the level of transcription regulation on target genes, promoting ectopic vulval induction in SynMuv mutants. The antagonistic roles of chromatin remodeling carried out by the SynMuv genes and the SynMuv suppressor genes are also involved in germline-soma distinction, RNAi, and other developmental events.

### Two Different NuA4-Like Complexes May Exist in C. elegans

The NuA4 complex is a well characterized S. cerevisiae HAT complex that acetylates the N-terminus of nucleosomal histones H2A and H4 and functions as a transcription coactivator [31]. The components of the human NuA4 complex correspond to a combination of two distinct complexes in yeast, namely NuA4 and SWR1 [31]. In this study, five SynMuv suppressor genes that we identified *(C34B7.4, gfl-1, mrg-1, Y105E8A.17,* and *ZK1127.3)* encode proteins homologous to components of the yeast NuA4 complex (Tables 1 and S1). C34B7.4 encodes a MYST family HAT enzyme that is a key component of the NuA4 complex in other species. In addition to *gfl-1,* and *Y105E8A.17,* three other SynMuv suppressor genes, *C08B11.6, C17E4.6,* and *CD4.7,* encode proteins homologous to components of the yeast SWR1 complex (Tables 1 and S1). The human homologs of six of these eight genes, *C17E4.6, C34B7.4, gfl-1, mrg-1, Y105E8A.17,* and *ZK1127.3,* encode components of the human NuA4 complex. Furthermore, it has been shown that the MRG-1 and ZK1127.3 proteins directly bind to each other in the yeast two-hybrid assay [39], implicating that these two proteins may function in the same complex in C. elegans. In fact, the human ortholog of MRG-1 directly binds to MRGB, a human ortholog of ZK1127.3 [30]. Thus, these observations allow us to consider that there is a NuA4-like complex in C. elegans that plays a similar transcription activation role to the yeast and human NuA4 complexes. Our findings that C34B7.4, GFL-1, MRG-1, ZK1127.3, and C08B11.6 are required for the ectopic expression of *lag-2* in the intestine of SynMuv B mutants (Table 3) further support the hypothesis that these proteins may act as transcription activators.

Intriguingly, it has recently been shown that strong *lf* mutations in three SynMuv C genes results in the Muv phenotype in SynMuv A or SynMuv B mutant backgrounds [7]. These three genes, *epc-1, mys-1,* and *trr-1,* also encode components of the NuA4 complex. MYS-1 may function as the MYST family HAT enzyme in a NuA4-like complex with EPC-1 and TRR-1, as suggested in a previous report [7]. The SynMuv phenotype indicates a repressive role of these genes in vulval induction. Consistent with this, *epc-1, mys-1, and trr-1* have been shown to repress the ectopic expression of *lag-2* in the intestine [18]. Therefore, the NuA4 complex containing these components appears to function differently from the above-proposed NuA4-like complex containing SynMuv suppressors that promote vulval induction. A repressive role of the NuA4 complex has also been observed in mammalian cells [72], suggesting that transcriptional repression by NuA4 complexes may be conserved between C. elegans and mammals. These results suggest that there may be two distinct NuA4-like complexes with opposing functions. An alternative explanation is that there is only one NuA4 complex with both positive and negative roles on transcription regulation in development. However, such a scenario is unlikely for the following reasons. In SynMuv A or SynMuv B mutants, RNAi of NuA4 components identified as SynMuv suppressors did not cause a Muv phenotype [7] (unpublished data), indicating that these genes do not have the same repressive roles as that of the SynMuv C genes. Reciprocally, RNAi of the three SynMuv C genes that are NuA4 components failed to suppress the SynMuv phenotype of *lin-15AB(n765)* (unpublished data), suggesting that these SynMuv C genes do not have the activation roles associated with the SynMuv suppressor genes. Biochemical purification and characterization of these complexes will elucidate whether there are two different NuA4-like complexes and what their actual functions are in C. elegans.

### The Role of Chromatin Remodeling in Transgene Silencing and RNAi

A number of SynMuv B genes are required for repetitive transgene expression in somatic cells. In contrast, we found that *dcr-1,* an RNAi pathway gene, as well as four SynMuv suppressor genes *(mes-4, isw-1, mrg-1,* and *zfp-1),* are required for the somatic transgene silencing at the transcriptional level. Since SynMuv B proteins are likely transcription repressors and SynMuv suppressor proteins are likely transcription activators, somatic transgene expression may be inhibited by factors or functions that are targets of these two classes of genes with opposite roles.

It has been known that tandem repeats form heterochromatin to silence gene expression [73]. Recent studies have revealed that components of the RNAi machinery are associated with the formation of heterochromatin [74]. Kelly and colleagues [75] have shown that histone H3 lysine 9 (H3K9) di- and trimethylation, a modification associated with heterochromatin and recognized by the heterochromatin protein HP1, is found on extrachromosomal transgenic fragments in C. elegans. It is consistent that the C. elegans HP1 homolog, *hpl-2,* and RNAi pathway genes are required for both germline transgene silencing and somatic transgene silencing associated with RNAi transcriptional gene silencing and *tam-1(lf)* [64,67] (unpublished data). It suggests that heterochromatin formation on extrachromosomal transgenes might be the major cause for transgene silencing in *C. elegans.*

We propose that SynMuv B genes and SynMuv suppressors play opposite regulatory roles on repetitive transgene silencing through regulating heterochromatin formation. One simple explanation for the hypersilencing associated with SynMuv B mutants is that the SynMuv B genes repress the expression of target genes involved in heterochromatin formation, while the SynMuv suppressor genes activate the expression of these target genes. In addition, the phenomenon of RNAi enhancement associated with the SynMuv B mutations may also be explained by the up-regulation of target genes in RNAi pathway in the SynMuv B mutants. We have so far been unable to identify the potential target genes. However, our efforts excluded the possibility of *hpl-2* and 11 RNAi pathway genes acting as the transcriptional targets of *lin-35/Rb* to regulate transgene silencing and RNAi, since the mRNA levels of these genes were not changed in *lin-35(n745)* mutants (Figure S1). To test such a hypothesis, an extensive genome-wide expression pattern analysis of SynMuv B and SynMuv suppressor genes may be necessary.

### Roles of SIN3 and SAM Synthetase in Vulval Differentiation Opposing to that of SynMuvB Protein

Identification of *pqn-28,* the C. elegans ortholog of the yeast/human SIN3 protein, as a SynMuv suppressor was unexpected because SIN3 generally functions as a transcription corepressor together with a histone deacetylase (HDAC). *hda-1,* encoding a worm HDAC, has been shown to repress the ectopic vulval induction [13,25], while we found in this study that *pqn-28* promotes ectopic vulval induction. Since there are several more genes in C. elegans genome that encode HDAC superfamily proteins, one simple explanation for this intriguing finding is that PQN-28 may interact with one or more of these HDAC proteins and function as a transcription activator. There have been genetic and genomic evidences that SIN3 may activate as well as repress transcription [49]. One alternative explanation is that PQN-28 may interact with transcription activators to regulate gene transcription independent of HDAC function in *C. elegans.*

Another interesting finding is that the gene encodes a SAM synthetase that also behaves as a SynMuv suppressor. In addition to suppressing the SynMuv phenotype, RNAi of this gene also rescued the larval lethality of *mep-1(lf)* and reverted the transgene silencing in the soma (Table 3). SAM can serve as a substrate for multiple histone methyltrasferases, including histone H3 lysine 9 (H3K9) and histone H3 lysine 4 (H3K4) methyltransferases. H3K9 methylation is known to be involved in transcription repression, while H3K4 methylation is known to be involved in transcription activation [28]. Therefore, it is possible that SAM synthetase is involved in both gene activation and repression. However, our genetic assay only revealed the SynMuv suppressor role that is likely involved transcription activation. One explanation is that the cellular concentration of SAM may be more critical to the H3K4 methylation than to the H3K9 methylation, with regard to its specific functions.

### Functional Distinctions among Different SynMuv Suppressor Genes

All 32 candidate genes were identified by suppression of the SynMuv phenotype, which indicates a common function for these genes in vulval induction. Although we expect that many of them act on one or more common targets for the function, not all of the suppressors behave the same. For example, *zfp-1(lf)* and *mes-4(lf)* have different genetic interactions with *let-60/Ras(gf)* mutations, indicating that these two genes do not act on the same targets for vulval functions. The functional differences among these SynMuv suppressor genes were also indicated by the differential roles of several cellular processes examined. For example, we showed that *isw-1* was required for RNAi and the ectopic expression of the *pgl-1* and *lag-2* genes, but was not essential for germline transgene silencing (Table 3). Three SynMuv suppressor genes in the protein degradation group *(C34D4.14, rpn-10*, and *rpn-12)* were determined to be involved in RNAi. However, RNAi of any of these three genes had no effect on rescuing the *mep-1(lf)* larval lethal phenotype, desilencing transgenes in the germline, or suppressing the ectopic expression of *lag-2* in the intestine (Table 3). Overall, these results implicate that there are functional distinctions among different chromatin remodeling factors even though they share some common functions.

### Conclusive Remarks

In this study, we identified and characterized a large number of potential chromatin remodeling genes that antagonize the functions of the SynMuv B pathways in several developmental and cellular processes. Our genetic analysis also implicated potential chromatin remodeling functions for several genes that do not reveal a connection to chromatin remodeling by structure alone. While each of these factors is implicated to play regulatory roles on many target genes, the study underscores the involvement of multiple chromatin remodeling complexes in regulating specific gene expression for respective functions. The precise mechanisms by which these chromatin-remodeling factors collaborate and coordinate to regulate gene expression and cellular functions remain to be investigated. Identifying target genes that are responsible for specific developmental or cellular functions, such as RNAi, vulval induction, and transgene silencing, would be the key to the success of further studies. As most of the SynMuv suppressor genes are conserved in mammals, genetic characterization of these genes in C. elegans should shed light on the role of the mammalian chromatin remodeling factors in development and related human diseases.

## Materials and Methods

### Genetic mutations and strains.

Information regarding the following mutations can be found at http://www.wormbase.org. LGI: *mes-3(bn35), dpy-5(e61), lin-35(n745),* and *sDP2(l,f).* LGII: *mes-2(bn11), unc-4(e120), lin-8(n111), lin-38(n751), rrf-3(pk1426),* and *mnC1.* LGIII: *lin-36(n766), lin-37(n758), lin-9(n112),* and *zfp-1(ok554).* LGIV: *eri-1(mg366), mep-1(q660), pgl-1(ct131), dpy-20(e1282), let-60(n1046), mes-6(bn66),* and *nT1(IV;V).* LGV: *mes-4(bn23)* and *dpy-11(e224).* LGX: *lin-15A(n767), lin-15B(n374), lin-15B(n744),* and *lin-15AB(n765). nT1(qIs51)* was used as dominant green balancer chromosomes. PD4251 (*ccIs4251; dpy-20(e1282)),* PD6249 *(ccIs4251; tam-1(cc567),* JK2868 *(unc-119(ed3); qIs56(lag-2::gfp, unc-119(+)),* and PD7271 *(pha-1(e12123ts); ccEx7271)* are described in previous reports [11,75–77]. The Bristol strain N2 was used as the WT strain.

### Genome-wide RNAi screen.

For the genome-wide screen, RNAi clones from the RNAi library [29] were inoculated in 96-well plates with 200 μl of Luria broth medium containing 50 μg/ml ampicillin and 15 μg/ml tetracycline per well, and cultured for 16 h at 37 °C. These RNAi clones were transferred to a new 96-well plate with 200 μl of Luria broth medium supplemented with 50 μg/ml ampicillin per well and again cultured for 16 h at 37 °C. NGM plates (6-cm) supplemented with 0.4 mM IPTG and 50 μg/ml ampicillin were seeded with these cultures, and were incubated for 24 h at room temperature and then spotted with four to eight synchronized L1 larvae of *lin-15AB(n765).* The plates were incubated for 7 d at 19 °C and then were scored for the Muv phenotype. Three rounds of retest were performed to determine the final list of positive suppressors. For the retests, RNAi clones were inoculated into 5 ml of Luria broth medium containing 50 μg/ml ampicillin and were cultured for 16 h at 37 °C. Each bacterial culture (~400 μl) was dispensed onto NGM plates supplemented with 0.4 mM IPTG and 50 μg/ml ampicillin. For all clones that suppressed the Muv phenotype of *lin-15AB(n765),* the dsRNA-encoding DNA inserts were sequenced. Several clones were found to contain genes that were different from those listed in the library. All RNAi screen were done at 19 °C except those specified.

### Other RNAi experiments.

An RNAi construct for *mes-2* gene is not present in the available RNAi library [29]. To make the RNAi feeding construct for *mes-2,* 1.5 kb of *mes-2* genomic DNA was PCR-amplified from genomic DNA and subsequently cloned into pPD129.36 (gift from A. Fire). The construct was transformed into Escherichia coli HT115(DE3). Injection RNAi was carried out as described previously [76]. Worms were injected with 200 ng/ml dsRNA of the candidate gene and were allowed to recover for 12–16 h before single worms were placed for egg-laying.

The co-injection experiments were conducted at 20 °C, as described previously [50,61]. DNA templates were PCR-amplified from plasmids corresponding to each RNAi clone with T7 promoters. dsRNA were synthesized in a single reaction using T7 polymerase-based transcription kit (Ambion Megascript Kit; Ambion, Austin, Texas, United States). Concentrations of dsRNAs were determined using a spectrophotometer. The quality and size of the dsRNAs were assessed by gel eletrophoresis. WT young hermaphrodites were co-injected with the mixture of 300 ng/μl of the dsRNA of candidate gene and 75 ng/μl of *mom-2* dsRNA. Worms were allowed to recover for 12–16 h before single worms per plate were placed for consecutive 48-h egg laying. Then, the total number of eggs was scored upon removal of P0 worms from the plates. The viability of the progeny were assayed after 24 h and expressed as the (number of hatched larvae) / (total number of eggs).

### Characterization of *zfp-1(ok554).*

The *zfp-1(ok554)* lesion was determined by sequencing genomic DNA and found to be identical to that reported by the C. elegans Gene Knockout Consortium (http://celeganskoconsortium.omrf.org) for the corresponding gene, *F54F2.2.* Primers outside of the deletion (attcaatcagcctgtggagg and tgctgctgctttctcgttta) and a primer inside of the deletion (gagggtccgcaagcatatgc) were used to confirm there is no additional copy of *zfp-1* in the genome and to distinguish the homozygotes from the heterozygotes when building double or triple mutants with mutations in other genes.

### Vulval induction assay.

VPC (P3p-P8p) cell fates in L4 hermaphrodites were scored under Nomarski optics as described previously [78]. Scores of 1, 0.5, and 0 were assigned to cells that fully, partially, or did not adopt vulval cell fates, respectively. In WT worms, three VPCs (P5.p, P6.p, and P7.p) fully adopted the vulval fate and the other three VPCs fused with epidermal syncytium, giving a score of 3.0. A score of more than 3.0 indicates ectopic VPC induction.

### Quantitative real-time RT-PCR (qRT-PCR).

Total RNA was isolated from worms using TRI Reagent (MRC, Inc., Cincinnati, Ohio, United States) followed by purification and DNase I treatment. Random-primed cDNA was prepared using Superscript III reverse transcriptase (Invitrogen, Carlsbad, California, United States). Reactions were run in triplicate with 2XSYBR Green Jumpstart Taq ReadyMix (Sigma, St. Louis, Missouri, United States) on the Rotor Gene 3000 (Corbett Research, Cambridge, United Kingdom). Relative fold changes were calculated using the 2^−ΔΔCt^ method. For *pgl-1,* we picked ~200 non-green L1/L2 stage worms (*mep-1(q660)* homozygote) from *mep-1(q660)/nT1(qIs51)* mothers treated with or without RNAi of the SynMuv suppressor genes. Primers for real-time PCR were designed to span an exon-exon junction. The *rpl-26* gene encoding the ribosome large subunit was used as an internal control for data normalization. For measuring the expression of *gfp/lacZ,* we picked ~200 L4-young adult stage worms from the strains PD4251, PD6249, and the progeny of the PD6249 injected with dsRNA of *dcr-1, isw-1, mes-4*, or *mrg-1*. RNA polymerase II large subunit AMA-1 gene was used as an internal control for data normalization. Primers for *ama-1* mRNA, mature mRNA of *gfp/lacZ,* and pre-mRNA of *gfp/lacZ,* were designed by Grishok et al. [64]. For measuring the mRNA levels of *hpl-2* and 11 RNAi pathway genes (see Figure S1) in *lin-35(n745),* synchronized L1 larvae are used. The *ama-1* was used as the internal control for data normlization. The data shown are representative of three experiments with independent worm growths and RNA isolations.

### Assay for the early larval arrest of *mep-1(q660)* rescue.

The *mep-1(q660)* mutant is temperature sensitive: at 25 °C, nearly 100% of *mep-1* homozygotes derived from a heterozygous mother are arrested as young larvae (L1 or L2) [79]. The RNAi plates were seeded with 12–18 synchronized L1 larvae of *mep-1(q660) IV/nT1(qIs51)* and cultured at 25 °C. *qIs51* is a genomic insertion containing three markers: *myo-2::GFP* expressed in the pharynx throughout development, *pes-10::GFP* expressed in the embryo, and *F22B7.9::GFP* expressed in the intestine [79]. Any *mep-1(q660)* homozygote hermaphrodite (nongreen animals) in the progeny were transferred onto the corresponding new RNAi plates. The larval progeny were scored four days later for larval stages according to the cell divisions of three VPCs (P5.p–P7.p) and the body size under Nomarski optics.

### Assay for somatic transgene silencing.

The restoration of green fluorescent protein (GFP) fluorescence in the strain PD6249 (*ccIs4251*; *tam-1(cc567)*) [11] was assayed for the GFP expression intensity by fluorescent microscopy with Nomarski optics, following feeding RNAi of all the candidates. The integrated array (*ccIs4251*) contains three markers: *myo-3* promoter driving mitochondrial targeted GFP, *myo-3* promoter driving a nuclear-targeted *GFP/LacZ* fusion gene, and a *dpy-20(+)* gene [76]. The RNAi plates were seeded with 12–16 synchronized L1 larvae and cultured for 6 days at 20 °C. The GFP expression in the F1 progeny at L4 to young adult stages was examined.

### Assay for germline transgene silencing.

Strain PD7271 (gift from W. Kelly) carries a multicopy extrachromosomal transgene array of *let-858::GFP,* of which the expression is silenced in the germline, but is expressed in most somatic lineages [75]. Synchronized L1 larvae of the strain PD7271 (12–18 larvae) were placed onto the candidate RNAi plates. The restoration of GFP expression in the germline was assayed in adults of the F1 progeny.

### Assay for *lag-2::gfp* ectopic expression in the intestine.

The ectopic expression of *lag-2::gfp* in *lin-15B(n744)* was assayed as described previously [25]. In brief, mid-L4 worms were mounted on 3% agar pads and were scored for the ectopic expression in the intestine cells under fluorescence microscope with Nomarski optics.

### Immunofluorescence staining.

PGL-1 antibody staining was conducted using L1 larvae that were fixed as described by Finney and Ruvkun [80]. Affinity purified polyclonal anti-PGL-1 antibody (gift from S. Strome) was used at 1:2000 dilution.

### Bioinformatics.

Analysis of the distributions of SynMuv and SynMuv suppressor genes in the TOPO expression mountains was performed using a program in http://workhorse.stanford.edu/cgi-bin/gl/gl_mod.cgi [70].

## Supporting Information

Figure S1 Quantification of Relative mRNA Levels of *hpl-2,* and 11 RNAi Pathway Genes in *lin-35(n745)* Mutant *rpl-26* was used as the internal reference. Mean values and ranges of the *pgl-1*/*rpl-26* ratios based on three qRT-PCR trials are shown.
(655 KB EPS)

Table S1 The 32 SynMuv Suppressor Genes and Their Homologs in Other Species (38 KB DOC)

Table S2 The Muv Suppression Observed in Two SynMuv Mutants upon RNAi of Each of the 32 Candidate Genes (38 KB DOC)

Table S3 The SynMuv B Mutants Enhanced Effect of Feeding RNAi (25 KB DOC)

## Abbreviations

dsRNA: double-stranded RNA
*gf*: gain of function
GFP: green fluorescent protein
HAT: histone acetyltransferase
HDAC: histone deacetylase complex
L1/L2: first or second larval
*lf*: loss of function
LZ: leucine zipper motif
MAPK: mitogen-activated protein kinase
NuA4: nucleosomal acetyltransferase of histone H4
NuRD: nucleosome-remodeling deacetylase
OM: octapeptide motif
PHD: plant homeodomain
RNAi: RNA interference
RP: regulatory particle
RTK: receptor tyrosine kinase
SAM: S-adenosylmethionine synthetase
SynMuv: synthetic multivulva
VPC: vulval precursor cell
WT: wild type
