# Supplementary material for: Diverse Chromatin Remodeling Genes Antagonize the Rb-Involved SynMuv Pathways in C. elegans
Source: PLoS Genet. 2006 May 19;2(5):e74. doi: 10.1371/journal.pgen.0020074 (PMC1463046; doi:10.1371/journal.pgen.0020074)
Supplement: Supplementary file 1 [file pgen.0020074.st001.doc]

# Supporting Information

**Table S1.** The 32 synMuv Suppressor Genes and Their Homologs in Other Species

| **Gene** | ***H.s.* ^a^** | ***D.m.* ^a^** | ***S.c.* ^a^** | **Gene Function/Domain** |
| --- | --- | --- | --- | --- |
| NuA4 complex | | | | |
| C34B7.4 | MYST3 | Enok | Esa1 | MYST histone acetyltransferase |
| *gfl-1* | YEATS4/GAS41 | CG9207 | Yaf9 | YEATS domain |
| *mrg-1* | MORF4L1 | Mrg15 | Eaf3 | Transcription factor MRG15 |
| Y105E8A.17 | DMAP1 | CG11132 | Swc4 | DNMT1-associated protein |
| ZK1127.3 | - | - | Eaf7 | Member of CT20 family |
| SWR1 complex | | | | |
| C08B11.6 | ACTR6 | Actr13E | Arp6 | Actin related protein 6 |
| C17E4.6 | TCFL1 | CG4621 | Vps72 | YL-1 protein |
| CD4.7 | ZNHIT1 | CG14038 | Vps71 | Hit zinc finger |
| COMPASS complex | | | | |
| C14B1.4 | WDR5 | Wds | Swd3 | WD-repeat protein 5 |
| *dpy-30* | LOC84661 | CG6444 | Sdc1 | Dosage compensation |
| *hcf-1* | HCFC1 | Hcf | Kel1 | Host cell factor C1 |
| *mes-4* | WHSC1L1 | Ash1 | Set2 | Set domain |
| ISW1 complex | | | | |
| *isw-1* | SMARCA1 | ISWI | Isw1 | Member of the ISW1 complex |
| SWI/SNF complex | | | | |
| *lss-4* | BAF250/p270 | Eld/Osa | Swi1 | Eld/osa, swi1 |
| Protein degradation | | | | |
| C34D4.14 | HECTD1 | CG5604 | Ufd4 | Ubiquitin-protein ligase |
| F30A10.10 | USP48 | Ubp64E | Ubp8 | Ubiquitin-specific protease |
| *rpn-10* | PSMD4 | Pros54 | Rpn10 | 26S proteosome subunit |
| *rpn-12* | PSMD8 | Rpn12 | Rpn12 | 26S proteosome subunit |
| Others | | | | |
| R08C7.3 | H2AFZ | His2Av | Htz1 | Histone H2A variant |
| T02C12.3 | GTF3C5 | CG10563 | Tfc1 | TFIIIC subunit |
| ZK856.9 | FLJ20729 | CG1463 | Bcd1 | TFIIIC subunit |
| F52B11.1 | CXXC1 | CG17446 | - | PHD domain |
| Y53G8AR.2 | PHF16 | Rno | Nto1 | PHD domain |
| B0035.4 | PFDN4 | CG10635 | Gim3 | Prefoldin subunit |
| *zfp-1* | MLLT10/AF10 | Alh | Nto1 | AF-10 protein |
| *pqn-28* | SIN3A | Sin3A | Sin3 | SIN3 homolog |
| C06E7.1 | MAT2A | M(2)21AB | Sam1 | S-adenosylmethionine synthetase |
| C34E10.8 | SRRM1 | - | - | Unknown function |
| F54D11.2 | FLJ10081 | CG8233 | - | Unknown function |
| K08F4.2 | G3BP | Rin | - | Unknown function |
| M03C11.3 | ACIN1 | CG12750 | - | Unknown function |
| T07E3.3 | LOC51064 | - | - | Unknown function |

^a^The predicted homologs in *Homo sapiens* (*H.s.*); *Drosophila melanogaster* (*D.m.*); *Saccharomyces cerevisiae* (*S.c.*); and a short description of their (putative) function and/or the domains.
