# Supplementary material for: Diverse Chromatin Remodeling Genes Antagonize the Rb-Involved SynMuv Pathways in C. elegans
Source: PLoS Genet. 2006 May 19;2(5):e74. doi: 10.1371/journal.pgen.0020074 (PMC1463046; doi:10.1371/journal.pgen.0020074)
Supplement: Supplementary file 2 [file pgen.0020074.st002.doc]

**Supporting Information**

***Table S2.*** *The Muv Suppression Observed in Two SynMuv Mutants Upon RNAi of Each of the 32 Candidate Genes*

| **Gene** | **Description** | **%Muv (n)**  ***lin-36; lin-15A* ^a^** | **%Muv (n)**  ***lin-8; lin-15B* ^a^** |
| --- | --- | --- | --- |
| RNAi control |  | 98 (338) | 76 (699) |
| NuA4 complex | | | |
| C34B7.4 | MYST histone acetyltransferase | 45 (240) ^*^ | 18 (347) ^**^ |
| *gfl-1* | Also in the SWR1 complex | 21 (280) ^**^ | 29 (772) ^**^ |
| *mrg-1* | Transcription factor MRG15 | 47 (343) ^*^ | 4 (401) ^**^ |
| Y105E8A.17 | Also in the SWR1 complex | 70 (235) ^*^ | 71 (177) |
| ZK1127.3 | Member of CT20 family | 8 (200) ^**^ | 2 (250) ^**^ |
| SWR1 complex | | | |
| C08B11.6 | Actin related protein 6 | 9 (252) ^**^ | 6 (1164) ^**^ |
| C17E4.6 | YL-1 protein | 60 (250) ^*^ | 36 (271) ^*^ |
| CD4.7 | Hit zinc finger | 55 (200) ^*^ | 40 (487) ^*^ |
| COMPASS complex | | | |
| C14B1.4 | WD-repeat protein 5 | 83 (184) | 28 (599) ^**^ |
| *dpy-30* | Dosage compensation | 63 (186) ^*^ | 35 (941) ^**^ |
| *hcf-1* | Host cell factor C1 | 40 (252) ^**^ | 24 (447) ^**^ |
| *mes-4* | Set domain | 7 (322) ^**^ | 2 (537) ^**^ |
| ISW1 complex | | | |
| *isw-1* | Member of the ISW1 complex | 2 (241) ^**^ | 2 (634) ^**^ |
| SWI/SNF complex | | | |
| *lss-4* ^b^ | Eld/osa, swi1 | 40 (200) ^**^ | 30 (320) ^**^ |
| Protein degradation | | | |
| C34D4.14 | Ubiquitin-protein ligase | 54 (347) ^*^ | 43 (612) ^*^ |
| F30A10.10 | Ubiquitin-specific protease | 49 (199) ^*^ | 37 (570) ^*^ |
| *rpn-10* | 26S proteosome subunit | 98 (248) | 42 (329) ^*^ |
| *rpn-12* | 26S proteosome subunit | 68 (292) ^*^ | 48 (466) ^*^ |
| Others | | | |
| R08C7.3 | Histone H2A variant | 6 (200) ^**^ | 13 (151) ^**^ |
| *zfp-1* | AF-10 protein | 55 (300) ^*^ | 36 (532) ^*^ |
| M03C11.3 | Chromatin factor | 7 (225) ^**^ | 4 (569) ^**^ |
| *pqn-28* | SIN3 homolog | 8 (172) ^**^ | 10 (382) ^**^ |
| B0035.4 | Prefoldin subunit | 76 (174) ^*^ | 61 (354) |
| C06E7.1 | SAM synthetase | 77 (164) ^*^ | 60 (800) |
| T02C12.3 | TFIIIC subunit | 94 (376) | 42 (497) ^*^ |
| ZK856.9 | TFIIIC subunit | 76 (157) ^*^ | 45 (374) ^*^ |
| F52B11.1 | PHD domain | 89 (356) | 51 (628) ^*^ |
| Y53G8AR.2 | PHD domain | 37 (261) ^**^ | 56 (374) ^*^ |
| C34E10.8 | Unknown function | 17 (224) ^**^ | 6 (578) ^**^ |
| F54D11.2 | Unknown function | 87 (323) ^*^ | 23 (1030) ^**^ |
| K08F4.2 | Unknown function | 34 (205) ^**^ | 31 (493) ^**^ |
| T07E3.3 | Unknown function | 28 (303) ^**^ | 37 (773) ^**^ |

^a^ The percent Muv was scored in *lin-36(n766)*; *lin-15A(n767)*, and *lin-8(n111)*; *lin-15B(n374)* following RNAi of each candidate genes. As RNAi control, the *E. coli strain* HT115 transformed with the empty vector pPD129.36 was used. All RNAi experiments were conducted at 19°C. *P<0.05. **P<0.01. P values were derived from comparing data from *lin-15AB(n765)* animals fed with RNAi of each candidate genes to those of *lin-15AB(n765)* fed with control RNAi using Fisher’s Exact Test.

^b^The data obtained with P0s, since RNAi of *lss-4* caused a complete sterile phenotype in P0s.
