# Supplementary material for: Diverse Chromatin Remodeling Genes Antagonize the Rb-Involved SynMuv Pathways in C. elegans
Source: PLoS Genet. 2006 May 19;2(5):e74. doi: 10.1371/journal.pgen.0020074 (PMC1463046; doi:10.1371/journal.pgen.0020074)
Supplement: Supplementary file 3 [file pgen.0020074.st003.doc]

**Supporting Information**

**Table S3.** The SynMuv B Mutants Enhanced Effect of Feeding RNAi

| Genotype | Phenotype scored upon feeding RNAi | | | | |
| --- | --- | --- | --- | --- | --- |
|  | *lin-1*  %Muv (n) | *hmr-1* | *dpy-13* | *rol-3* | *unc-33* |
| N2 | 0 (240) | Weak BMD | Dpy (+) | WT | WT |
| *rrf-3(pk1426)* | 75 (396) | P0s: sterile | Dpy (+++) | Rol (+++) | Unc (++) |
| *eri-1(mg366)* | 67 (488) | P0s: sterile | Dpy (+++) | Rol (+) | Unc (++) |
| *lin-9(n112)* | 83 (71) | P0s: sterile | Dpy (+++) | Rol (+++) | Unc (++) |
| *lin-15B(n744)* | 89 (170) | P0s: sterile | Dpy (+++) | Rol (++) | Unc (+) |
| *lin-35(n745)* | 88 (128) | 100% BMD | Dpy (++) | Rol (+) | WT |
| *lin-36(n766)* | 0 (200) | weak BMD | Dpy (+) | WT | WT |
| *lin-37(n758)* | 65 (74) | 100% BMD | Dpy (+++) | Rol (+) | Unc (+) |
| *tam-1(cc567)* | 27 (252) | 100% BMD | Dpy (++) | Rol (+) | WT |
| *lin-8(n111)* | 0 (210) | Weak BMD | Dpy (+) | WT | WT |
| *lin-38(n751)* | 0 (200) | Weak BMD | Dpy (+) | WT | WT |

The phenotypes assayed were Muv for *lin-1(RNAi)*, Sterile or BMD (Body Morphogenesis Defects) for *hmr-1(RNAi)*, Dpy (Dumpy) for *dpy-13(RNAi)*, Rol (Roller) for *rol-3(RNAi)*, and Uncoordinated (Unc) for *unc-33(RNAi)*.WT: wild type other than the phenotypes associated with the strains themselves. The symbols of +, ++, and +++, indicate the severity of the phenotype.

**Figure S1.** Quantification of Relative mRNA Levels of *hpl-2*, and 11 RNAi Pathway Genes in *lin-35(n745)* Mutant. *rpl-26* was used as the internal reference. Mean values and ranges of the *pgl-1*/*rpl-26* ratios based on three qRT-PCR trials are shown.
